# Ocular neuromodulation as a novel treatment for retinitis pigmentosa: identifying rod responders and predictors of visual improvement

**DOI:** 10.1186/s40942-025-00699-w

**Published:** 2025-07-01

**Authors:** Ismail M Musallam

**Affiliations:** Musallam Eye Center of Jerusalem, Beit Hanina, Jerusalem, Israel

**Keywords:** Retinitis pigmentosa, Ocular neuromodulation, Low luminance questionnaire-10, Rod responders, Ascorbic acid, Substance P

## Abstract

**Purpose:**

To evaluate the safety and efficacy of ophthalmic nerve stimulation (ONS), combined with ascorbic acid (AA) in the treatment of retinitis pigmentosa (RP).

**Methods:**

Forty participants with RP were enrolled in a prospective open-label single-armed intervention. Patients with non-syndromic RP; aged ≥ 4 years, with BCVA ≥ 20/400 were included. All participants were treated with bilateral ONS sessions combined with intravenous administration of AA for two weeks. The primary efficiency endpoint was the change in scotopic vision at 6 months, assessed using 10-item, 100-point, Low Luminance Questionnaire (LLQ-10). The secondary efficiency points included BCVA and contrast sensitivity. Rod responders were defined by ≥ 25 points increment of LLQ-10 score at 6 months’ visit.

**Results:**

Ocular neuromodulation therapy significantly improved low luminance vision, BCVA, and contrast sensitivity in patients with RP (*p ≤ 0.05*). At 6-month visits, 60% of patients were identified as rod responders. The mean change in LLQ-10 score was (46.35 ± 16.81 point) in rod responders versus (4.9 ± 7.6 point) in non-responders (*p < 0.0001*). A clinically significant improvement of BCVA (≥ 0.2 logMAR unit) and contrast sensitivity (≥ 0.3 log unit) were demonstrated in 50% of the right eyes of rod responders.

**Conclusion:**

Ocular neuromodulation significantly improved night vision, BCVA, and contrast sensitivity. Determinants of rod responders include the duration of night blindness, stage of the disease, and thickness of ganglion cell layer at baseline. Two therapeutic scenarios were recognized; an early disease-modifying intervention that restores night vision and a late cone rescue intervention that improves/maintains central vision.

## Introduction

Retinitis pigmentosa (RP) is a group of inherited eye conditions, affecting 1 in 3000 people, with most patients becoming legally blind by the age of 40. It is characterized by progressive loss of rods followed by cone photoreceptor death, and abnormal hemodynamics of the eye [1].

RP is not merely caused by genetic factors; in fact, it is a complex multifactorial disorder. Other factors such as reduced ocular blood flow (OBF) are assumed to contribute to its pathogenesis. Evaluating choroidal hemodynamic changes has shown the reduction in choroidal blood flow (ChBF) is proportional to the progression of RP [2, 3]. Recently, relative choroidal ischemia and reduced ChBF have been demonstrated in RP by measuring the choroidal vascularity index via Spectral Domain Optical Coherence Tomography (SD-OCT) [4]. The OBF is reduced not only in the retina [5] and choroid but also in the retro-ocular vessels [6]. Interestingly, signs of reduced OBF have been observed before the appearance of any of the fundoscopic features of RP [7].

Elevated levels of endothelin-1 were observed in the plasma of RP patients [6]. Endothelin-1 is a potent vasoconstrictor peptide that is synthesized and released by endothelial cells. Increased endothelin levels can lead to a predominance of vasoconstricting factors associated with a decrease in OBF and vascular dysregulation which might play a primary role in the pathogenesis of RP [1].

Ophthalmic artery and its main branches including ciliary and choroidal arteries are all under both parasympathetic and sympathetic neural control with many also under local trigeminal control [8]. The parasympathetic input has a vasodilatory effect, while the sympathetic input has a vasoconstrictor effect [8, 9].

**Fig. 1:**
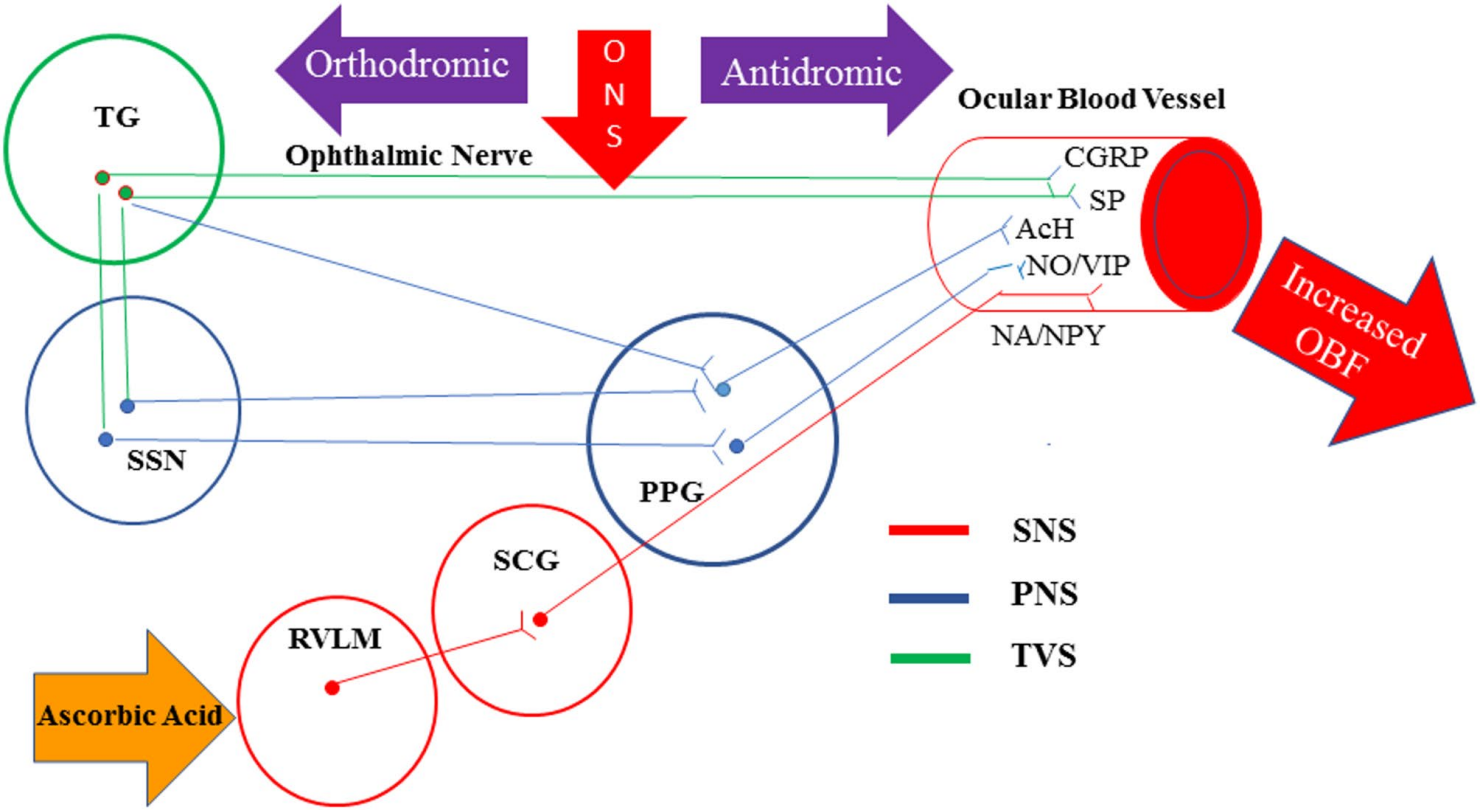
Schematic of neural pathways that control ocular blood flow. SNS; sympathetic nervous system, PNS; parasympathetic nervous system, TVS: trigeminovascular system, TG: trigeminal ganglion, PPG: pterygopalatine ganglion, SSN: superior salivatory nucleus, SCG: superior cervical ganglion, RVLM: Rostral ventrolateral medulla, SP: substance P, CGRP; calcitonin gene-related peptide, Ach: acetylcholine, NO: nitric oxide, VAP: vasoactive intestinal polypeptide, NA: noradrenaline, NPY; neuropeptide Y

Stimulation of sensory afferents from the ophthalmic nerve results in the activation of three pathways that lead to vasodilatation and increased OBF (Fig. 1). The first is the activation of the trigeminovascular system via antidromic impulse [8, 9]. The nasociliary nerve, which originates from the ophthalmic nerve, contains major vasodilatory innervation [10, 11], and its stimulation results in the release of vasoactive.

neuropeptides from the free nerve endings, such as substance P (SP) and Calcitonin gene related peptide (CGRP) [12]. The second pathway results in parasympathetic vasodilation of the ocular vasculature via interactions with the facial nerve and sphenopalatine ganglion [13, 14]. The third pathway involves a central activation of the rostral ventrolateral medulla which induces vasodilation and subsequent increase in OBF [15]. Consistent with this, stimulation of the ophthalmic nerve in experimental animals was found to increase OBF [16], along with enhanced uveal release of SP [17]. Overexpression of endogenous SP by ONS is capable of boosting both the OBF [8, 9, 16] and tissue regeneration by the recruitment of endogenous stem cells [18]. Moreover, SP can protect diverse types of cells including retinal pigment epithelium [19], and ganglion cells [20]. It also contributes to the prevention of apoptosis [21], and suppression of both oxidative stress [22] and inflammation [23, 24].

Oxidative stress plays a central role in the pathogenesis and progression of RP. Anti-oxidant interventions have been reported to substantially delay photoreceptor cell death [25]. AA is highly concentrated in the retina [26]. The therapeutic, non-enzymatic scavenging of free radicals can be accomplished by AA, but only at supraphysiological concentrations [27]. To achieve this concentration in the retina, the intravenous route might be ideal to bypass tight control of oral administration.

Based on the effects of ONS, AA, and the functioning of SP investigated in previous studies, we hypothesized that neuromodulation-based therapy for RP may be capable of recovering the functions of dormant rods and cones and preventing their death. The study aims to evaluate the safety and efficacy of ONS paired with intravenous administration of ascorbate for treating participants with RP. Additionally, baseline predictive factors for night vision improvement were also defined.

## Materials and methods

Forty participants with RP were recruited into a prospective, open-label single-armed interventional study, conducted at a single Eye Center. This study was approved by the Institutional Review Board, and it adhered to the tenets of the Declaration of Helsinki. Written informed consent was obtained from all participants or legal guardians after an explanation of the purpose and possible outcome of therapy.

**Fig. 2:**
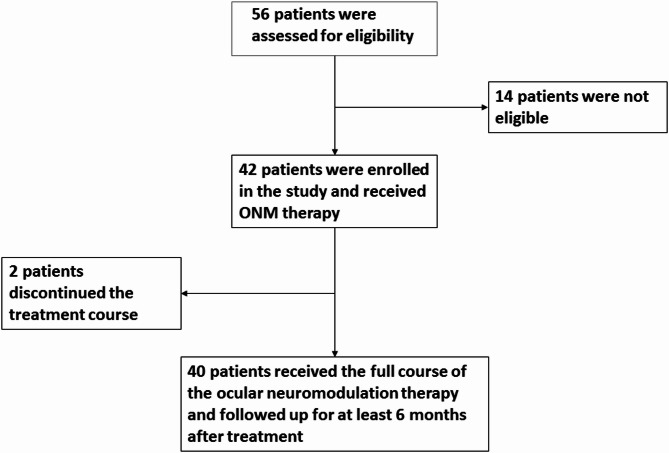
Sample Size, Enrollment, and Study Design: Among the 56 patients who were assessed for eligibility, 14 patients were excluded from participating in the study. These include; 9 who have Usher syndrome, 2 who have Bardet Biedl syndrome, and three who met the criteria for exclusion due to advanced stage of the disease in both eyes (stage V with BCVA ≥ 20/400). Additionally, two other patients were excluded from the study as they did not receive the full course of the treatment (5 sessions out of 12 sessions). 4 patients in the cohort had only one eligible eye. In one patient, the other eye was excluded because of vision loss due to long-standing retinal detachment, while in the other 3 patients, the other eye was excluded because of poor fixation and advanced stage of the disease (stage V with BCVA ≥ 20/400)

All ophthalmological examinations, staging of the disease, and patient selection were performed by a senior vitreoretinal specialist (the author). Figure 2, shows details of the participant’s enrollment.

Following recruitment, all subjects with a medical history and established diagnosis of RP completed baseline vision testing, slit lamp examination, and fundoscopy. Spectral Domain Optical Coherence Tomography (SD-OCT) was performed within 2–4 weeks before treatment. The severity of the disease was clinically graded into six stages based on fundoscopy and SD-OCT findings (Table 1).

**Table 1:** Staging of RP

Stage	Clinical Description
**Stage 0**	Patients experience no symptoms related to retinitis pigmentosa. Fundus examination reveals a normal-looking retina with no pigmentary changes, no visible retinal blood vessel changes, or optic nerve pallor. Classic electroretinogram findings for retinitis pigmentosa are detected
**Stage 1**	Patients may experience mild night vision disturbance. Fundus examination reveals a normal-looking retina with no pigmentary changes, no visible retinal blood vessel changes, or optic nerve pallor. Classic electroretinogram findings for retinitis pigmentosa are detected
**Stage II**	Mild attenuation of retinal blood vessels that extend anteriorely beyond the mid periphery of the retina but not reaching ora serrata, no or mild optic disc pallor, bone spicule pigmentary changes are limited to mid-periphery of the retina.
**Stage III**	Moderate attenuation of retinal blood vessels that extend anteriorely up to mid-periphery with mild optic nerve pallor. Pigmentary changes are located at mid-periphery and extend centrally reaching the vascular arcade.
**Stage IV**	Severe attenuation of retinal blood vessels ended with the formation of a complete vascular arcade, combined with moderate optic nerve pallor. Pigmentary changes are becoming more diffuse and extend centrally to the inside of the vascular arcade, but sparing the macula. Central retinal thickness is less than 150 µ as measured by Spectral Domain Optical Coherance Tomography
**Stage V**	Very severe attenuation or complete obliteration of retinal blood vessels that ended at a short distance forming incomplete vascular arcade (≤ 4 disc diameter) from optic nerve disc. Optic pallor is remarkable and the macula is involved in pigmentary changes. Stage V is also coined to any retina with a central retinal thickness of 100 μm or less as measured by Spectral Domain Optical Coherance Tomography

For Sample size estimation the online One Arm Binomial program was used [28], with the power of 90% and $\:\propto\:$= 0.05 with an expected proportion of responders of 25%, the minimal sample was estimated to be at least 30 patients ( Fig. 2). Inclusion criteria were: patients with an established diagnosis of classic, non-syndromic RP; aged ≥ 4 years, with BCVA ≥ 20/400. Exclusion criteria were syndromic RP pregnancy, glaucoma, central retinal vein occlusion, and retinal detachment.

To evaluate the therapy outcome, low luminance questionnaire, (LLQ-10) score changes from baseline to 6 months after treatment were calculated (The primary outcome measures). Given the importance of patient-centered outcomes in clinical trials [29, 30], LLQ-10 serves as a valuable tool for assessing visibility issues in reduced light levels. LLQ-10, derived from the validated LLQ-32 questionnaire [31], was refined to prioritize items specific to low-luminance vision in RP patients (Table 2). Rod responders were defined by ≥ 25 points increment of LLQ-10 score at 6 months after treatment.

**Table 2:** Low luminance Questionnaire-10 (LLQ-10)

Q	Subject	Question Text
1	Night Vision	Rate how much difficulty you have seeing at night.
2	Peripheral vision	Rate how much difficulty you have in your peripheral vision at night.
3	Reading in dim light	Rate how much difficulty you have in reading menus in dimly lit restaurants or reading the newspaper/book without good lighting.
4	Depth of Perception	Rate how much difficulty you have in-depth perception at night.
5	Color Vision	Rate how much difficulty you have seeing colors at night.
6	Home life	Rate how much difficulty you have seeing furniture in dimly lit rooms.
7	Social life	Rate how much difficulty you have going out to nighttime social events such as sports events, the theater, friend’s homes, church, mosque, or restaurants?
8	Parties in dim light	Rate how much difficulty seeing in candlelight?
9	Dependency on others	Rate how often you depend on others to help you because of your vision at night or under poor lighting?
10	Psychological Stress	Rate how often you are worried or concerned that you might fall at night because of your vision?

**Scoring Instructions**: Each of the 10 questionnaire items should be answered by one of the following: (1) No difficulty at all (10 points). (2) Little difficulty (7.5 points). (3) Some or moderate difficulty. (5 points). (4) A lot of difficulties (2.5 points). (5) Completely blind under these conditions (0 points). The total score on the LLQ-10 ranges from 0 to 100 points, with a higher score indicating better vision under low luminance. The scotopic vision difficulties are further classified into four levels of dysfunction according to the LLQ-10 score. Level 4; (0–25) which indicates absolute night blindness /Minimal scotopic vision, level 3 severe night blindness (> 25–50), level 2; moderate night blindness (> 50–75) and level 1 (> 75–100) with almost normal scotopic vision. Rod responders were identified as those in whom the LLQ-10 score increased by ≥ 25 points at 6 months’ post-treatment.

The secondary efficiency points include BCVA and contrast sensitivity of the right eye. These visual functions were evaluated under standardized conditions by a single well-trained optometrist who was not aware of the previous results of the participant. BCVA was recorded in Snellen equivalents and then transformed into a logMAR unit. Contrast sensitivity was measured using a Pelli- Robson chart under standardized photopic illumination at one meter in each eye, using best correction spectacles. For safety, purposes, ocular and non-ocular adverse events were reported.

The ocular neuromodulation protocol included the administration of AA followed by ONS over a period of two weeks. AA was given intravenously in a dose of 3 gm. dissolved in 100 ml of saline infused at 5 ml min^− 1^ for 20 min on the first day, followed by a drip infusion of 1 gm. daily during the rest of the treatment period.

**Fig. 3:**
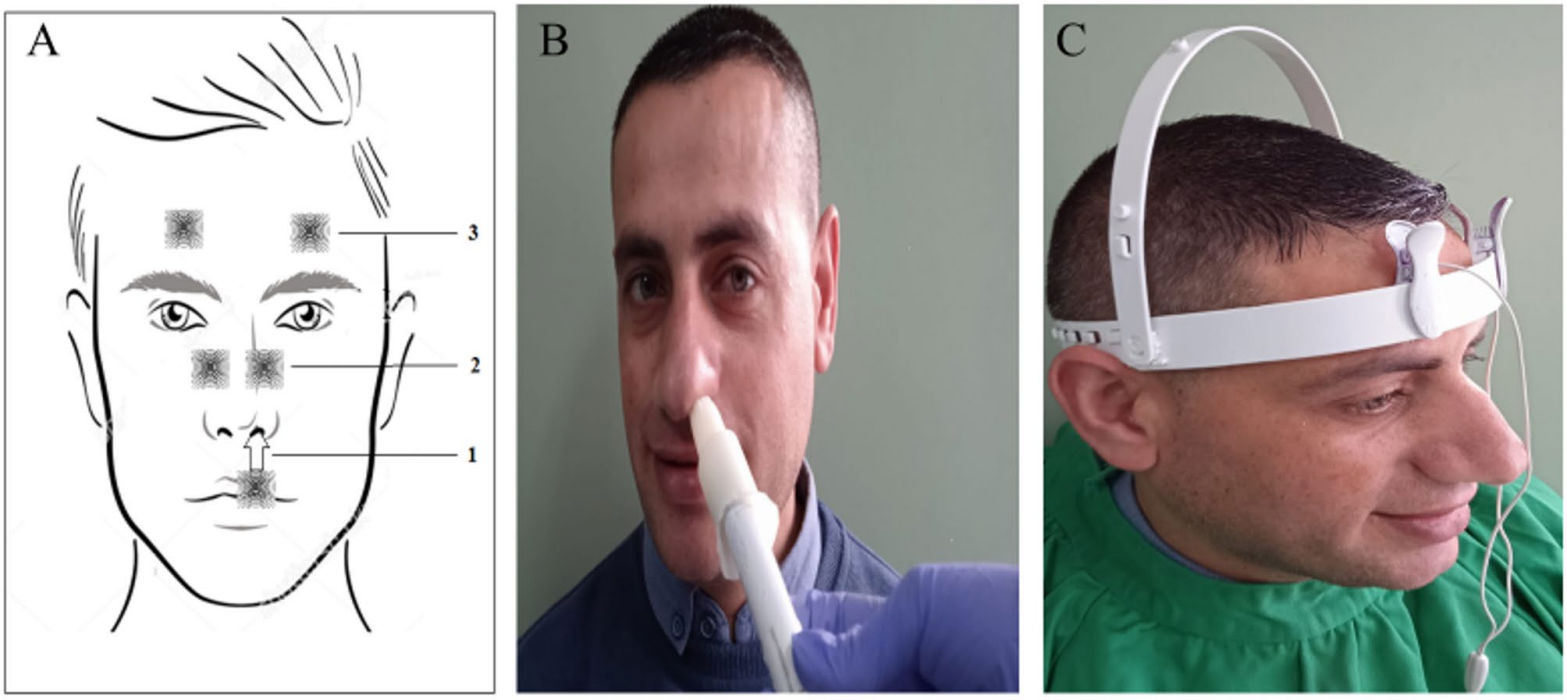
(**A**) Sites of ophthalmic nerve stimulation(ONS): (1) Intranasal: for stimulation of anterior ethmoid nerve at the nasal septum, (2) Nasal bridge: for stimulation of the infratrochlear nerve, a branch of nasociliary nerve and external nasal nerve, the terminal branch of external anterior ethmoid nerve, (3) Supraorbital region: for stimulation of supraorbital and supratrochlear nerve, branches of frontal nerve. (**B**) Handheld vibrotactile ophthalmic nerve stimulator. An intranasal application head is designed to be small enough to fit comfortably and safely within the restricted size of the nasal vestibule and optionally made by polyethyl methacrylate to prevent the warming produced by the vibration. (**C**) Head-mounted vibrotactile ophthalmic nerve stimulator with two application heads over the supraorbital region on both sides

A modulated low magnitude, low-frequency vibration, with a frequency of approximately 60–90 Hz and stimulation amplitude range of 1.5–3.5 μm was used for ONS. This setting was adapted to selectively activate the parasympathetic system and trigeminovascular system [8, 9, 32]. Modulated frequency and amplitude waveforms were used to avoid adaptation to mechanical stimulus. Hand-held and head-mounted prototypes of ophthalmic nerve stimulators were used to activate different stimulation sites (Fig. 3) by using modified commercially available micro-vibrators, along with different types of intranasal and extra-nasal application heads.

Bilateral ONS was applied to the subjects over a session of 30 min per day, 6 sessions per week, and one day off. Each session included intra-nasal vibrochemical (20 min), and extra-nasal vibrotactile stimulation over the nasal bridge (5 min) and the supraorbital region in both sides (5 min) (Fig. 3A), using appropriate application heads. During intra-nasal vibrochemical stimulation, the nasal application head was covered by a rubber cape impregnated with 2% menthol cream (Dermacool plus ^R^) as a transient receptor potential cation channel subfamily M (melastatin) member 8 (TRPM8) agonist (Fig. 3B). Participants were assessed clinically, and by SD-OCT, and ILQ-10 at baseline and at 1, 6, 12, and 24 months after treatment.

The statistical analysis was performed using SPSS^R^, V. 21. The mean differences in the characteristics of the rod responders and rod-non-responders were compared using the independent sample t-test. The change in the mean value of visual function parameters between baseline and different post-treatment points was evaluated using a paired sample t-test. A non-parametric test (Mann-Whitney U test) was used to compare the median value of different parameters comparing the rod responders and rod-non-responders. The change in the median value of visual function parameters between baseline and different post-treatment points was evaluated using the Wilcoxon signed-rank test. The relationship between the stage of the disease and rod responsiveness was evaluated using the Chi-square test. Additionally, the longitudinal data of the secondary outcomes (BCVA and Contrast sensitivity) during the 24 months after treatment were analyzed using linear mixed-effects models to assess the changes in each outcome. The BCVA and contrast sensitivity at baseline, and at post-treatment visits were recorded for each eligible right and left eye in the whole cohort. Specifically, for each outcome variable, the mean of the outcome was modeled as a linear function of time since baseline, and a main effect for cohort and an interaction effect between cohort and time was also included in the modeling.

Multivariate logistic regression was performed to study the factors that independently predict rod responders and to control for confounding factors. All tests were two-tailed and a *p-value* less than 0.05 was considered significant.

## Results

Between April 14, 2018, and April 29, 2020, 40 participants, aged between 4 and 55 years were included. The inheritance mode was autosomal recessive in 21 participants, and autosomal dominant in 2, and sporadic in 17 participants. The demographics, baseline characteristics, and determinants of rod responders are presented in Table 3.

**Fig. 4:**
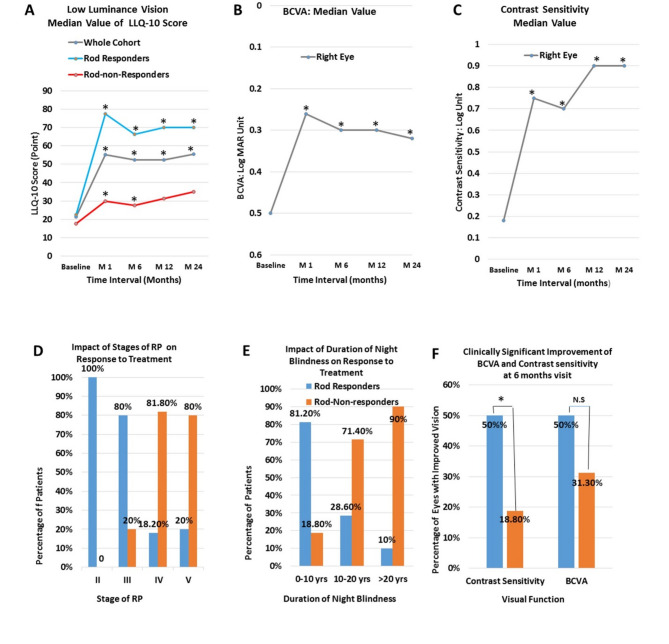
Changes in visual functions and predictors for rod responders: (**A**) Median value of LLQ-10 (**B**) The median value of BCVA in the right eye (**C**) The median value of contrast sensitivity in the right eye (**D**) Proportion (%) of rod responders and rod-non-responders based on stages of the disease. (**E**) Proportion (%) of rod responders and rod-non-responders based on durations of night blindness (**F**) Proportion (%) of right eyes of rod responders versus rod-non-responders that showed a clinically significant improvement of BCVA and contrast sensitivity (* p ≤ 0.05), N.S; Not significant)

Twenty-four (60%) patients were identified as rod responders. Table 4 shows the mean (mean ± SD) and the median value (median; 25th, 75th ) of LLQ-10 score at different intervals after treatment. Figure 4A shows the median value of the low luminance vision score at different post-treatment intervals. In the whole cohort, the median score of LLQ-10 improved significantly from 21.3 points at baseline to 55.1 points (*p = 0.0001*) at one month, 52.5 points (*p = 0.0001*) at 6 months, and 52.5 points (*p = 0.0001*) at 12 months and to a value of 55.5 points (*p = 0.001*) at 24 months after treatment.

**Table 3:** Baseline characteristics and determinants of rod responders

Character	Total cohort	Rod Responders	Non-rod Responders	*P* Value
**Participant Number**	40	24 (60%)	16 (40%)	
**Age: yrs.**				
Mean ± SD Median (median; 25th,75th ) **Range**	28.4 ± 14.1 24.0 (19,40) 4–55	23.9 ± 12.2 21.5 (16.5,32.0) 4–49	35.1 ± 14.3 40.00 (21.3,47.0) 7–52	0.02 0.015
**Gender; Male/Female (males %**)	24/16 (60%)	13/11 (54.2%)	10/6 (62.5%)	0.6
**Duration of night blindness (yrs.)**				
Range Mean ± SD Median	2–46 16.2 ± 12.3 16.0	2–30 10.9 ± 6.7 10.0	3–46 24.2 ± 14.6 21.00.	0.0001 0.003
**Duration of Night Blindness**				
0–10 10–20 >20	16 (100%) 14 (100%) 10 (100%)	13(81.2%) 4 (28.6%) 1 (10%)	3 (18.8%) 10 (71.4%) 9 (90%)	
Stage of the Disease *				
II, Number of patients (%) III, Number of patients/ (%) IV, Number of patients (%) V, Number of patients/ (%)	9 (100%) 15 (100%) 11 (100%) 5 (100%)	9 (100%) 12 (80%) 2 (18.2%) 1 (20%)	0 (0%) 3 (20%) 9 (81.8%) 4 (80%)	0.0001
**LLQ-10 Score**				
Mean ± SD Median (median; 25th,75th )	23.1 ± 13.0 21.3 (12.5, 34.4)	23.6 ± 11.6 22.5 (15.0,34.4)	22.2 ± 15.2 17.5 (12.5,34.4)	0.73 0.5
**BCVA**: logMAR				
Mean ± SD Median (median; 25th,75th )	0.54 ± 0.35 0.50 (0.20,0.80)	0.44 ± 0.33 0.34 (0.2,0.6)	0.69 ± 0.33 0.8 (0.5,1.0)	0.03 0.03
**Contrast Sensitivity**: log unit				
Mean ± SD Median (median; 25th,75th )	0.43 ± 0.48 0.18 (0,0.9)	0.57 ± 0.50 0.7 (0,0.9)	0.25 ± 0.38 00 (0, 0.6)	0.074 0.056
**Central retinal thickness**: µm				
Mean ± SD Median ( 25th,75th )	215.1 ± 64.4 203.0 (175.5,261.0)	231.3 ± 59.9 224.0 (184.0,270.0)	188.5 ± 64.6 185.5 (134.8,221.3)	0.05 0.04
**Nerve Fiber Layer Thickness**: µm				
Mean ± SD Median ( 25th,75th )	42.4 ± 11.3 41.0 (35.3,47.8)	43.1 ± 12.7 41.0 (35.0, 48.3)	41.2 ± 8.9 41.0 (35.3, 48.3)	0.63 0.95
**Ganglion Cell Layer Thickness**: µm				
Mean ± SD Median ( 25th,75th )	59.9 ± 16.8 57.0 (50,70.8)	65.4 ± 16.2 60.0 (51.0, 77.0)	50.2 ± 13.4 49.0 (40.5, 55.5)	0.0001 0.002

At the 6-month visit, the low luminance vision score was significantly improved in the whole cohort by (29.8 ± 24.7) points as compared to baseline (*p < 0.0001).* The mean change in LLQ-10 score was (46.35 ± 16.81) in rod responders versus (4.9 ± 7.6) in non-responders (*p = 0.0001*).

The baseline characteristics and determinants for rod responders are shown in Table 3. Multivariate logistic regression indicated that the rod responsiveness was predicted by duration of the night blindness (*p* = 0.047), stage of the disease (*p* = 0.05), and ganglion cell layer thickness (*p* = 0.03). Upon consideration of the disease stage (Table 3; Fig. 4D), it was observed that individuals exhibiting rod responsiveness were predominant in the earlier stages of the disease (*p* = 0.0001). Notably, all patients in stage II (comprising 9 individuals) and 80% of patients in stage III (12 out of 15) demonstrated rod responsiveness. (Table 3; Fig. 4E). Furthermore, most patients (81.3%) (13 out of 18) who experienced night blindness for 10 years or less were found to be rod responders. (Fig. 4E).

**Table 4:** Visual functions at various post-treatment intervals

Character	Baseline	1 month	6 months	12 months	24 months
**LLQ-10 score (Whole Cohort):**					
(Mean ± SD) P value	23.1 ± 13.0	55.1 ± 28.6 (*p = 0.0001*)	52.8 ± 27.2 (*p = 0.0001*)	52.9 ± 27.4 (*p = 0.0001*)	56.5 ± 28.0 (*p = 0.0001*)
Median Value (median; 25th, 75th ) P value	21.3 (12.5,34.4)	55.1 (37.5,85.0) (*p = 0.0001*)	52.5 (37.5,85.0) (*p = 0.0001*)	52.5 (30.6,82.5) (*p = 0.0001*)	55.5 (32.5,85.0) (*p = 0.001*)
**LLQ-10 score (Rod Responders)**:					
(Mean ± SD) P value	(23.6 ± 11.6)	(73.5 ± 16.6) (*p = 0.0001*)	(70.0 ± 16.2) (*p = 0.0001*)	(69.7 ± 20.5) (*p = 0.0001*)	(68.9 ± 23.7) (*p = 0.0001*)
Median Value (median; 25th, 75th ) P value	22.5 (15.0,34.4)	77.5 (57.5,89.4) (*p = 0.0001*)	66.3 (53.8,86.9) (*p = 0.0001*)	70.0 (52.5,86.9) (*p = 0.001*)	70.0 (54.0,90.0.0) (*p = 0.002*)
**BCVA (Whole Cohort**,** RE) (Log Mar)**					
(Mean ± SD) P value	0.54 ± 0.35	0.33 ± 0.29 (*p* = 0.0001)	0.36 ± 0.30 (*p* = 0.0001)	0.40 ± 0.34 (*p* = 0.013)	0.37 ± 0.29 (*p* = 0.03)
Median Value (median; 25th, 75th ) P value	0.50 (0.02, 0.80)	0.26 (0.03, 0.52) (*p = 0.0001*)	0.3 (0.04, 0.6) (*p = 0.0001*)	0.3 (0.16, 0.6) (*p = 0.006*)	0.32 (0.12, 0.57) (*P = 0.03*)
**Contrast Sensitivity (whole cohort RE)**					
(Mean ± SD) P value	(0.43 ± 0.48)	(0.71 ± 0.51) (*p = 0.0001*)	(0.65 ± 0.53) (*p = 0.0001*)	(0.71 ± 0.55) (*p = 0.002*)	(0.70 ± 0.53) (*p = 0.02*)
Median Value (median; 25th, 75th ) P value	0.18 (0.0, 0.9)	0.75 (0.21, 1.25) (*p = 0.0001*)	0.7 (0.03, 1.25) (*p = 0.0001*)	0.9 (0.0, 1.25) (*p = 0.003*)	0.9 (0.0, 1.22) (*p = 0.02*)

When the BCVA is considered, the mean and median values of BCVA of the right eye of the whole cohort were significantly improved at all post-treatment visits when compared to baseline (Table 4; Fig. 4B).

At 6 months’ visit, 42.5% of the right eyes of the whole cohort demonstrated a clinically significant improvement of BCVA (improvement of ≥ 0.2 log MAR unit). Such improvement was demonstrated in 50% of the right eyes of rod responders compared to 31.3% of the right eyes of rod-non-responders (Fig. 4F).

The whole cohort (both eyes) had statistically significant improvement in the estimated marginal mean BCVA at one (*p* = 0.0001) and 6 months (*p* = 0.01) after treatment as it was measured by a linear mixed effects model. However, this improvement was not statistically significant at 12 and 24 months’ visits.

As regards to the effect of neuromodulation on contrast sensitivity, the mean, as well as median values of contrast sensitivity of the right eye of the whole cohort, were significantly improved at all post-treatment visits when compared to baseline. (Table 4; Fig. 4C).

At the 6-month visit, 37.5% of the right eyes of the whole cohort demonstrated a clinically significant improvement in contrast sensitivity (improvement of ≥ 0.3 log unit). Additionally, there was a statistically significant difference in the proportion of right eyes that showed a clinically significant improvement of contrast sensitivity among the rod responders compared to that of rod-non-responders (*p < 0.03*). 50.0% of the eyes of rod responders and 18.8% of the eyes of rod-non-responders had a clinically significant improvement in contrast sensitivity (Fig. 4F).

We also analyzed the therapeutic effects of ocular neuromodulation on the right and left eye separately by considering the change of BCVA and contrast sensitivity over time starting from baseline, up to 24 months, using the Friedman test for related samples. The results were significant for the right eye (*p = 0.0001*), as well as for the left eye (*p = 0.0001*) when the BCVA and contrast sensitivity were considered (*p = 0.0001*), This indicates that ocular neuromodulation significantly improves BCVA and contrast sensitivity in both eyes in a simultaneous fashion.

The whole cohort (both eyes) had statistically significant improvement in the estimated marginal mean contrast sensitivity at one (*p* = 0.002) and 6 months (*p* = 0.047) after treatment as measured by the linear mixed-effects model. However, this improvement was not significant at 12 and 24 months’ visits.

Clinically significant regression was noticed in 6 (11.8%) patients 6–12 months after the treatment. At 24 months’ visit, 20 out of 22 participants (91%) showed signs of visual regression that justified re-treatment. No serious adverse effects were reported and headache in 5 patients was the only encountered side effect in this study.

## Discussion

The current study reported promising results after the use of ocular neuromodulation as a novel treatment for RP that was previously considered incurable. It has led to clinically meaningful and statistically significant improvements in visual functions including scotopic vision, BCVA, and contrast sensitivity. We have identified patient characteristics that may predict a positive response to treatment. These include early stage of the disease, short duration of night blindness and ganglion cell layer thickness at baseline.

In this study, we found that the most robust improvement in visual function for the subjects with early stages of the disease was night vision as measured by LLQ-10. The process of rod cell apoptosis occurs over years [33], suggesting that there was a much wider window of opportunity for applying ocular neuromodulation for the early rod-related stage of RP. Recovery of night vision was likely to be related to the reactivation of dormant/starving rods via enhanced oxygen and glucose delivery and shunting metabolites toward aerobic glucose metabolism [34]. A recent study has demonstrated a protective effect of restored glucose transport to mutant rods in RP [35].

The pathophysiology of RP is complex and involves multiple pathways, including a variety of pathological vascular and cellular mechanisms. Therefore, effective treatment of RP requires targeting several factors. These include dysregulated OBF, oxidative stress, neuroinflammation [36], and neurovascular degeneration of the retina. All of these pathological changes are anticipated to be counteracted by ocular neuromodulation therapy, including the early normalization of abnormal retinal vasculature. This abnormality is induced by reduced levels of vascular endothelial growth factor (VEGF) [37], which progressively decrease with disease advancement, and by the upregulation of the endothelin system [6].

One validated mechanism of vascular normalization under such conditions is the enhancement of VEGF signaling. The angiogenic effect resulting from the indirect activation of the vagus nerve [38]—via its neuronal connection with the ophthalmic nerve [39] —might contribute to this process. Furthermore, sensory neurons of the ophthalmic nerve may also promote retinal angiogenesis through SP signaling in response to antidromic ONS [40].

**Fig. 5:**
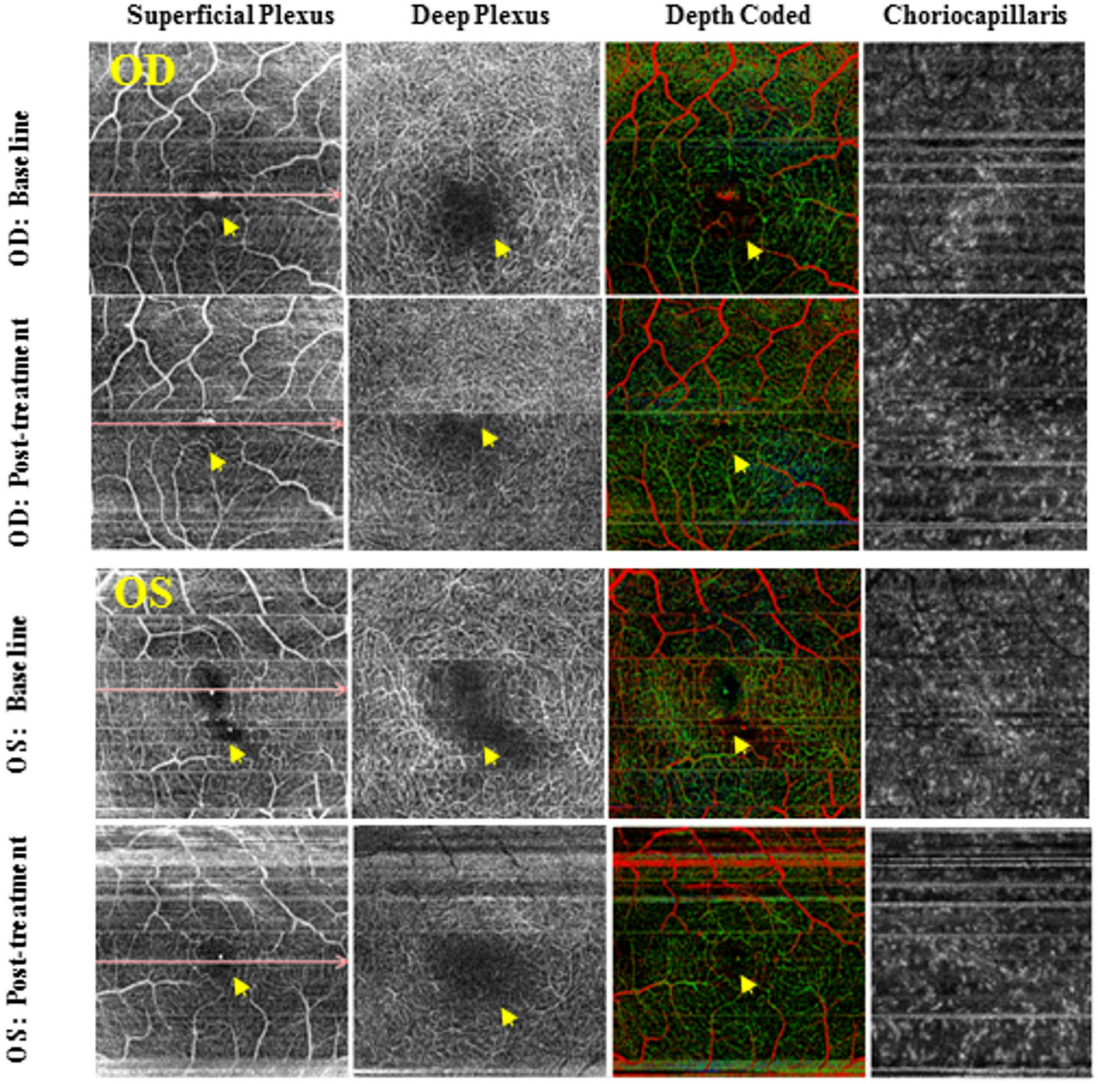
Vasculotrophic Effect of ONS-Based Therapy. OCT Angio of OD and OS of a six-year-old boy with stage II RP at baseline (pre-treatment) and 26 months’ post-treatment. The vascular density increased significantly at the para-foveal region at the level of superficial and deep capillary plexus in both eyes. The foveal avascular zone gets smaller after treatment. Yellow arrows indicate the site of non-perfused/drop out of capillaries at the level of the superficial and deep retinal plexus before and changes after treatment

Interestingly, the long-lasting vasoconstrictor effects of endothelin-1 can be mitigated by endogenously expressed CGRP [41]. Therefore, overexpression of CGRP via ONS may represent a novel strategy to treat endothelin-1–associated vasoconstriction and ocular vascular dysregulation. Additionally, SP expression might orchestrate endogenous stem cell recruitment for the regeneration of damaged neurovascular tissues in the retina [18, 40, 42]. Based on these mechanisms, we hypothesize that ONS promotes OBF and angiogenesis in the retina through the upregulation of VEGF, SP, and CGRP.

Evidence of improved OBF following ONS via a trans-corneal approach has been documented in studies involving healthy subjects without RP [43]. Transcorneal electrical stimulation in patients with RP has also been associated with increased OBF and significant vision improvement [44]. Although OBF was not directly measured in the current study, angiogenesis was observed—suggesting that ocular neuromodulation may facilitate remodeling of retinal blood vessels. This process, known as vascular normalization, aims to restore vascular structure and function. Nevertheless, we propose that the appearance of a normalized retinal vascular phenotype (Fig. 5) can only be achieved if ocular neuromodulation is initiated as early as possible—prior to the onset of irreversible vascular remodeling. This therapeutic window is herein referred to as the “vascular normalization window.”

The specific molecular anti-inflammatory events that occur in the retina as a result of ocular neuromodulation therapy remain to be established. One possible explanation for the reduction in retinal inflammation is improved ocular hemodynamics which results in the attenuation of retinal hypoxia and ischemia at the cellular level [45]. Significant reductions of TNF-α and IL-6 levels in response to ONS have also been reported [45]. On the other hand, SP has been demonstrated to suppress inflammation in different experimental settings by promoting M2 polarization [24, 46]. Then up-regulation of SP in the outer retina via ONS could offer anti-inflammatory effects by M2 polarization on the degenerating retina.

**Fig. 6:**
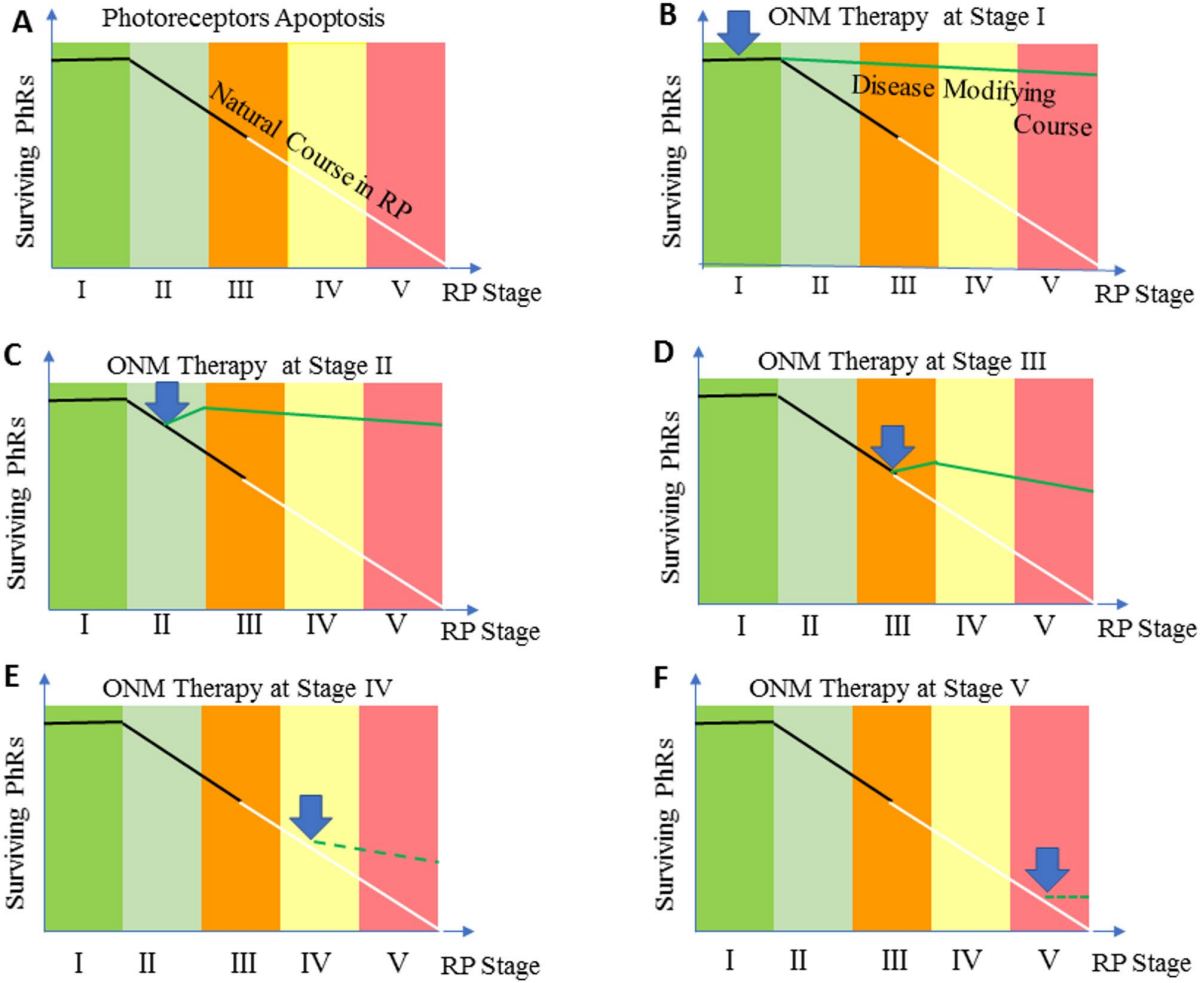
Photoreceptors degeneration and the effect of ocular neuromodulation therapy on the course of the disease: (**A**) The natural course of rods (black line) and cones (white line) degeneration in RP. The blue arrow indicates the time of initiation of ocular neuromodulation. (**B**) and (**C**) ocular neuromodulation therapy initiated at the early rod degeneration phase (stage I and II) respectively. The green line represents an early disease-modifying effect of ocular neuromodulation therapy to reverse the disease process via restoration of the function of dormant rods. (**D**) ocular neuromodulation therapy initiated at the transitional phase (stage III). The green line represents the anticipated modified course of rod and cone photoreceptors. In (**E**), and (**F**), the interrupted green line represents a cone rescue strategy wherein ocular neuromodulation therapy is initiated at stages, IV and V respectively (Late cone degeneration phase of RP)

In this study, the onset of therapeutic effects appeared within days after treatment, while the peak of its action took 2 weeks to reach, suggesting that some rewiring and/or circuit stabilization were developed after ocular neuromodulation. Following late intervention, almost no scotopic vision can be restored. Accordingly, two therapeutic scenarios of ocular neuromodulation have been recognized; an early disease-modifying intervention that might prevent or reverse the disease process and a late cone rescue intervention that aims to improve/maintain central vision (Fig. 6). The duration of action of ocular neuromodulation therapy was maintained for up to 24 months in rod responders (Fig. 4A, B, and C). Nevertheless, the long-term follow-up indicated that ocular neuromodulation would lose some of its efficacy at some point. Therefore, follow-up and re-treatment are mandatory to achieve efficacy, such that the visual improvements are maintained and effects do not diminish over time.

AA was used in the present study as a potentiating agent for ONS therapy. Initially a high dose of AA was given to prevent the oxidative stress caused by reperfusion of a hypoperfused retina and chronic retinal hyperoxia induceced by rod-depletion [47]. Prior to treatment—particularly following extensive rod dormancy or rod cell death—the retina exhibits reduced oxygen consumption. This leads to a hyperoxic environment that exacerbates oxidative stress [47]. As rod function gradually recovers and oxygen consumption increases, oxidative stress diminishes, and lower doses of AA become sufficient. In addition to its antioxidant role, AA inhibits the enzyme neutral endopeptidase [48], which degrades SP [49], thereby prolonging SP activity. Of note, the half-life time of SP is very short, from seconds to minutes [50].

Although AA is a potential confounder, current evidence does not support its efficacy as a monotherapy for RP. For instance, Komeima et al. (2006) found no significant therapeutic effect of high-dose AA monotherapy in an RP mouse model [51]. The dose used in the present study (14–42 mg/kg) is considerably lower. Nevertheless, given the single-arm design of the study, the contribution of AA cannot be entirely ruled out.

The treatment protocol in the current study was designed following detailed analysis, aiming to avoid the technical and procedural challenges reported in previous experiments. Prior clinical studies have evaluated the effects of electrical stimulation on RP [52–54]. These studies employed a range of electrical stimulation settings and targeted various sites, including transcorneal, transdermal, and transorbital stimulation, but achieved only limited improvement in vision [52–54]. This limited efficacy is likely due to the poor spatial resolution of electrical stimuli, progressive desensitization to prolonged suprathreshold stimulation and the delayed timing of intervention.

Identifying the optimal stimulation parameters, and paradigms that yield maximal visual recovery is a critical step in the translation of ONS for the treatment of RP. The intra-nasal route as a stimulation site has multiple advantages, including the presence of numerous mechanoreceptors and chemoreceptors, which are specifically distributed throughout the nasal mucosa, as shown by electrophysiological studies [55]. In the present study, administration of ONS intra- nasally is ideal as it is located closer to the all-important targets to be stimulated. This include anterior ethmoid nerve, sphenopalatine ganglion, and mid‐brain area where much of the autonomic functions lie. Vibrochemical ONS is emerging as a promising and potentially superior alternative to traditional electrical stimulation. It has the potential to upregulate both the ocular parasympathetic system and the trigeminovascular system [8, 9, 32]. Hiraba et al. utilized vibrotactile stimulation settings of 89 Hz frequency and 1.9 μm amplitude to selectively activate the parasympathetic system [32], which in turn leads to vasodilation and increased ocular blood flow [13, 14].

The co-localization of SP and TRPM8 in the slowly adapting nociceptive C fibers and mechanoreceptive Aδ fibers presents an ideal opportunity for the selective activation of SP/CGRP-loaded nasociliary nerve, a major division of the ophthalmic nerve [10]. This selective activation can be achieved intra-nasally through low-frequency mechanical stimulation and chemical stimulation using a TRPM8 agonist (menthol) [56]. SP overexpression triggered by ONS offers multiple retinal benefits, including enhanced blood flow, RPE proliferation, reduced apoptosis, stem cell recruitment, and inflammation suppression [8, 9, 16, 18–24].

The interpretation of study findings should be considered within the context of few limitations. These include small number of participants, lack of information regarding the genotype, OBF and retinal electrophysiology. There were no controls in this prospective interventional study. Alternatively, single-arm trials may be considered, especially in scenarios where the disease course is well known and patient pool is limited as in RP. While placebo effects cannot be entirely excluded, their impact is typically transient. Our findings demonstrated sustained improvements in visual functions up to 24 months’ post-treatment, especially in rod responders. These long-term effects argue against a placebo-driven response, particularly given RP’s progressive nature. Additionally, patients with early-stage RP (Stage 2) showed notable night vision improvements, whereas patients in later stages (Stages 4–5) did not, further suggesting a structure-function relationship. Nevertheless, this is a long-term, open-labeled single-armed intervention, and was intended to be an exploratory one. In the future, a proper control group should be considered in a large multicenter double-blind prospective study.

In conclusion, this study introduces ocular neuromodulation as an affordable, universal, and non-invasive treatment for various types of retinitis pigmentosa (RP), regardless to the genetic background. Its therapeutic effects are likely mediated by increased OBF, suppression of inflammation and oxidative stress, and the recruitment of endogenous stem cells through the overexpression of SP. To monitor treatment response effectively, biomarkers related to inflammation, OBF, and ocular SP—such as tear film SP—should be evaluated. These biomarkers are essential for assessing treatment efficacy, detecting disease progression, and guiding adaptive treatment strategies.

## Data Availability

The datasets used and/or analyzed during the current study are available from the corresponding author upon reasonable request.

## Abbreviations

AA: Ascorbic acid
Ach: Acetylcholine
BCVA: Best corrected visual acuity
CGRP: Calcitonin gene-related peptide
ERG: Electroretinogram
LLQ-10: Low Luminance Questionnaire-10
logMAR unit: Log of Minimum Angle of Resolution
M1: Microglia cell 1
M2: Microglia cell 2
NA: Noradrenaline
NPY: Neuropeptide Y
ONS: Ophthalmic nerve stimulation
PNS: Parasympathetic nervous system
PPG: Pterygopalatine ganglion
RP: Retinitis pigmentosa
RVLM: Rostro-ventrolateral medulla
SCG: Superior cervical ganglion
SD-OCT: Spectral Domain Optical Coherence Tomography
SNS: Sympathetic nervous system
SP: Substance P
TG: Trigeminal ganglion
TRPM8: Transient receptor potential cation channel subfamily M (melastatin) member 8
TVS: Trigeminovascular system
VAP: Vasoactive intestinal polypeptide
